# The Effect of Autologous Platelet Concentrates on Maxillary Sinus Augmentation: A Meta-Analysis of Randomized Controlled Trials and Systematic Review

**DOI:** 10.1155/2020/7589072

**Published:** 2020-06-15

**Authors:** Yusheng Meng, Xingxing Huang, Min Wu, Xiuqiao Yang, Yun Liu

**Affiliations:** Stomatology Health Care Center, Shenzhen Maternity and Child Healthcare Hospital Affiliated to Southern Medical University, Shenzhen 518048, China

## Abstract

**Introduction:**

To assess the efficacy of the autologous platelet concentrates (APCs) combined with autologous bone or bone substitute for the maxillary sinus floor lifting by a meta-analysis.

**Materials and Methods:**

Electronic databases (PUBMED, Web of Science, EMBASE through OVID, and Cochrane Library) were searched until Dec 31, 2019, and only randomized controlled trials (RCTs) in English were identified. Outcome variables included histologic evaluation, the implant stability quotient values, and radiographic evaluation. Data were analyzed by Revman5.3; the estimate of effect sizes was expressed as the 95% confidence interval; and the risk of bias was evaluated using the Cochrane Collaboration tool.

**Results:**

11 RCTs involving 141 patients (214 sites) were included in our meta-analysis, which indicated that the differences in the percentage of contact length among newly formed bone (2.61%, 95% CI, -1.18% to 7.09%), soft tissue area (-0.15%, 95% CI, -0.54% to 0.24%), and residual bone substitute material (-5.10%, 95% CI, -10.56% to 0.36%) in the APC group lacked statistical significance. Besides, there was the same effect on the implant stability quotient (ISQ) values of APC group who underwent implant placement 4 months after sinus augmentation and control group who received implant placement 8 months after sinus augmentation (-0.48, 95% CI, -1.68 to 0.72). No significant effect of APCs on the bone density was found (1.05%, 95% CI, -1.69% to 3.82%).

**Conclusions:**

The use of APCs in sinus augmentation may be further shorten the time required for bone graft maturation and allow earlier implant placement, but cannot enhance the bone formation in the long term. It is not currently recommended for routine use APCs as an osteoinductive material to bone grafting in sinus augmentation.

## 1. Introduction

Due to the aesthetic requirements of the patient and/or functional problems, one of the big challenges for implant dentistry is atrophic maxilla, which arises from the deficiency of hard and soft tissues [1]. Tooth loss in the posterior maxilla induces a progressive resorption of the alveolar bone, pneumatization of the maxillary sinus, and periodontal disease, making it very hard to place dental implants without the support of fixed prosthetic rehabilitation [2, 3]. Transalveolar osteotome sinus floor elevation is also called the osteotome technique, capable of increasing the density of the soft maxillary bone and elevating the sinus membrane with grafting substitute materials [4–6]. There are different kinds of osteoconductive materials to elevate the maxillary sinus floor, including autografts, freeze-dried bone allografts (FDBA), deproteinized bovine bone mineral (DBBM) xenografts, and alloplastic materials (hydroxyapatite, HA, and beta-tricalcium phosphate, *β*-TCP) [7]. However, these materials may cause the new bone formed to lack osteoinductive properties and osteogenic capacities, prolong the healing time, and trigger immune responses, etc. Some osteoinductive materials have none of these disadvantages and can enhance bone regeneration, such as autologous platelet concentrates (APCs), bone morphogenetic protein-2 (rhBMP-2), and mesenchymal stem cells (MSCs).

The APCs are prepared by the centrifugation of autologous blood, and three generations of APCs have been developed so far, including platelet-rich plasma (PRP), platelet-rich fibrin (PRF), and concentrated growth factors (CGFs) [8]. The PRP, as the first generation of APCs, was introduced into the field of stomatology by Marx in 1998 [9]. The concept of PRF, a new second generation of platelet concentrate, was raised by Choukroun in 2006 [10], and it is greatly different from PRP. Compared with PRP, there was no chemical additive added for the separation of PRF, and PRF could avoid the possibility of immune rejection and allergic reactions for its three-dimensional molecule network enabled a favorable cell migration, attachment, and differentiation. CGF is the latest generation of APCs introduced by Rodella et al. in 2006 [11], which contains a higher concentration of growth factors and releases growth factors and matrix proteins slowly. In order to verify the capability of APCs' osteoinductive effect on sinus augmentation with osteoconductive materials, we carried out a systematic literature review and meta-analysis on the efficacy of APCs in sinus augmentation.

## 2. Materials and Methods

### 2.1. PICOS Question

Our study was conducted and reported according to the PRISMA (Preferred Reporting Project Guidelines for Systematic Review and Meta-analysis) protocols [12]. The following statements were used to conduct a systematic search. The participants (P) included healthy adults with sinus augmentation and/or implants; the intervention (I) was APCs combined with osteoconductive materials for sinus augmentation; the comparison (C) was conducted without the APCs; the outcomes (O) comprised radiographic, histomorphometric, clinical, and postoperative implant stability quotient assessment; the study (S) was designed for humans, and only randomized controlled trials (RCTs) were enrolled.

### 2.2. Search Strategy and Terms

A computer-assisted literature search of the PUBMED, Web of Science, EMBASE through OVID, and Cochrane Library electronic databases was performed (last access Dec 31, 2019) to identify studies on the use of the APCs in sinus augmentation. The focus was on the hypothesis that the APCs could have a potentially positive effect on bone regeneration. The search strategy was performed by using the following terms: (“platelet concentrates” OR “platelet rich plasma” OR “platelet rich fibrin” OR “concentrated growth factors”) AND (“maxillary sinus augmentation” OR “maxillary sinus floor lift” OR “maxillary sinus floor elevation”). An additional hand search of the following journals was performed on the official websites: The International Journal of Oral & Maxillofacial Implants, Clinical Oral Implants Research, Journal of Dental Research, Journal of Prosthetic dentistry, and Journal of Prosthodontics. The final search was conducted on Dec 31, 2019.

### 2.3. Study Selection and Inclusion/Exclusion Criteria

The research that met the following criteria was included:

1. Randomized controlled trials (RCTs) on healthy adult patients aged over 20 years and over 4 months follow-up
2. Studies having a clear description of the histological and clinical results that revealed the additional effect of APCs on sinus floor augmentation
3. Studies presenting data about radiographic outcomes on the addition of APCs in sinus augmentation
4. Studies with the implant stability quotient after sinus augmentation evaluated

The exclusion criteria were as follows:

1. Studies involving patients with systemic contraindication or acute maxillary sinusitis or affected by uncontrolled periodontal diseases
2. Studies with incomplete data
3. Retrospective, prospective cohort studies, case reports, conference proceedings, and case series
4. Duplicate studies

### 2.4. Study Selection and Data Extraction

The titles and abstracts of the references in the databases were selected independently by two reviewers (Liu and Huang), who also performed the full-text reading of possible relevant articles. All disagreements were resolved by discussion and the third author (Wu) was consulted for consensus. Articles that did not meet the inclusion criteria were excluded, and the reasons for exclusion were recorded. Characteristics of the studies including author; publication year; study design; duration; number of patients; sex; mean age of the patients; intervention; clinical and radiographic observations; complications; radiography; histological results consisting of new bone formation, newly formed bone, and bone substitute; percentage of residual bone graft; soft tissue area; and the ISQ were also extracted.

### 2.5. Assessment of Risk of Bias in Included Studies

The risk of bias of comparative studies was evaluated independently by two authors (Liu and Huang) according to the standard of the Cochrane Handbook for Systematic Reviews of Interventions (Version 5.3). Any disagreement was resolved through discussion during the process. The considered items included selection bias (the sequence generation), selection bias (allocation concealment), performance bias, detection bias, attrition bias, reporting bias, and other biases. Plausible risk of bias for the parameters was assessed as adequate, unclear, or inadequate. The authors of the included studies were contacted and asked to provide explanations or missing information as needed, and a consensus was reached after discussion with them. A study was considered at low risk of bias when all items were met or one criterion was not adequate, moderate risk of bias if two items were not adequate, and high risk of bias if more than two parameters were judged to be inadequate.

### 2.6. Statistical Analysis

The meta-analysis was performed by Review Manager 5.3 (Cochrane Collaboration, Oxford, UK), and then a mean and a standard deviation were calculated using the method of Hozo et al. [13]. The data were expressed as mean difference (MD) and 95% confidence interval (CI). Besides, we also calculated the standardized mean differences (SMD) for different measures of outcome and gave their definitions. Heterogeneity was interpreted as recommended by the Cochrane Handbook, and *χ*^2^ and Higgins index (*I*^2^) were used to judge whether there was heterogeneity. The fixed effects model (FE) was used for meta-analysis if the heterogeneity between studies was low (*I*^2^ ≤ 50%), and the random effects model (RE) was employed if the heterogeneity between studies was significant (*I*^2^ > 50%). The heterogeneity between studies in radiographic, histomorphometric, and ISQ assessment was compared through subgroup analysis. Furthermore, the related forest plots were also generated.

## 3. Results

**Figure 1:**
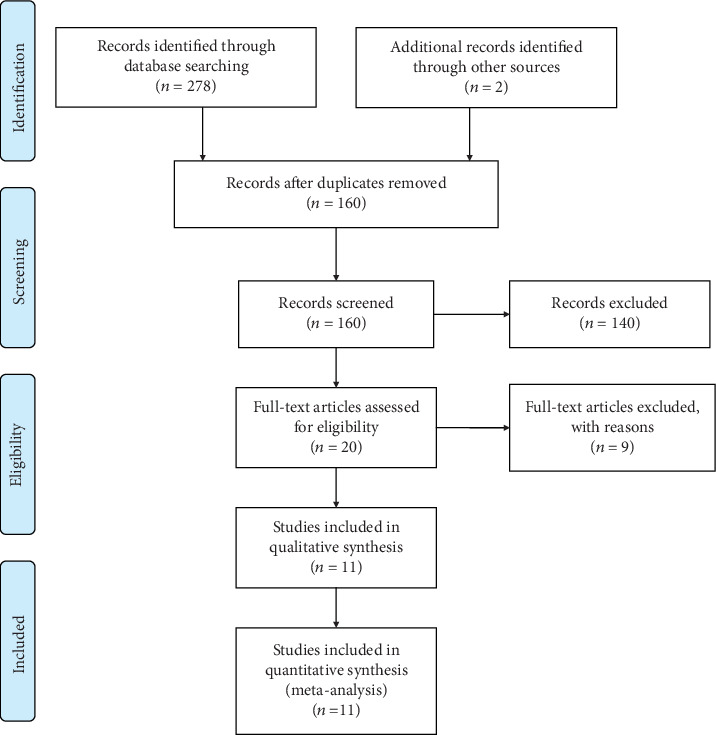
PRISMA flow diagram illustrating the selection process.

The article selection process is shown in Figure 1. A total of 300 articles were identified by the outline and hand searching. 160 records remained after the exclusion of duplicates, and 20 potential studies that met the eligibility criteria were selected. After full-text reading of these 20 articles, 11 articles were finally included for meta-analysis [14–24], and the other 9 articles were excluded because of the incomplete results. Finally, a total of 141 patients with 214 maxillary sinuses under treatment were enrolled, including 87 patients with 102 maxillary sinuses in PRF group and 54 patients with 112 maxillary sinuses in PRP group. Four articles adopted a parallel design and seven articles adopted a split-mouth design. The main characteristics and outcome data of the included studies are shown in Tables 1 and 2, respectively. The sensitivity analysis of results is shown in Table 3.

### 3.1. Quality Assessment of the Studies

**Figure 2:**
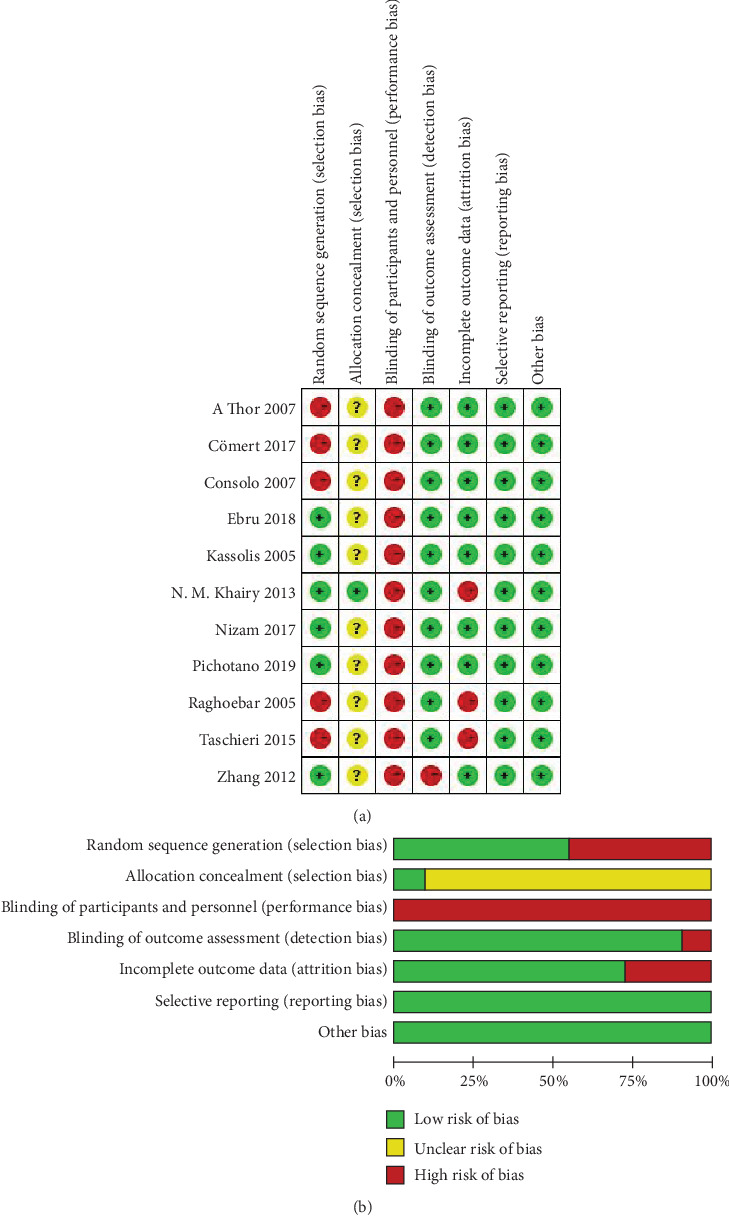
Risk of bias of the included studies.

The quality analysis of the comparative studies for risk of bias is shown in Figure 2. Five articles [17, 18, 21–23] were highly risky in sequence generation for they did not explain the methods of random generation. Allocation concealment was considered an unclear risk of bias in 10 articles because the method of allocation concealment was elevated, and only one article [19] was at low risk of bias for the investigator responsible for enrolling participants, who did not know in advance group, and the next person would join in the allocation concealment process. All performance biases were at high risk since the patients and operator were not made blind in the surgery. All the articles were at low risk of detection bias except one [20] without blinded operators in radiographic and histologic analyses. The risk of attrition bias was high in three articles because the bone biopsy of 2 ruptured sinuses was not feasible for histomorphometric analysis [18], and the postoperative pain and oedema were not evaluated [19], and one implant was removed [23]. The reporting bias and other biases of all articles were at low risk. In the end, five articles [14–16, 19, 24] were classified as moderate risk (two criteria were not met or unclear) and six articles [17, 18, 20–23] as high risk (three or four criteria were not met or unclear).

### 3.2. Analysis of Outcome Data

#### 3.2.1. Histologic Evaluation

**Figure 3:**
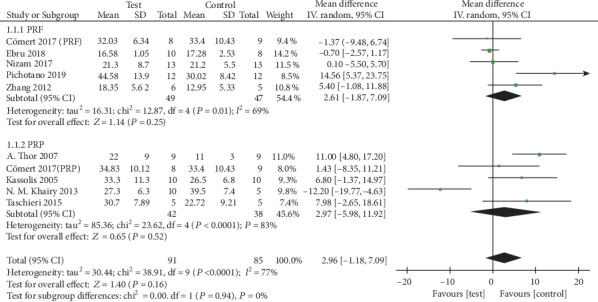
Percentage of new bone formation.

*(1) Percentage of New Bone Formation*. The meta-analysis was performed in nine studies [14–21, 24]. The random effects model (RE) was used (*I*^2^ = 77%). The results showed that APCs exerted 2.96% less new bone formation when added to osteoconductive materials in the maxillary sinus augmentation compared with osteoconductive materials alone (Figure 3), but there was no significant difference (95% CI, -1.18% to 7.09%; *p* = 0.16). The subgroup analysis revealed also no significant difference for PRF and PRP, with an MD of 2.61% (95% CI, -1.87% to 7.09%; *p* = 0.25) and an MD of 2.97% (95% CI, -5.98% to 11.92%; *p* = 0.52), respectively.

**Figure 4:**
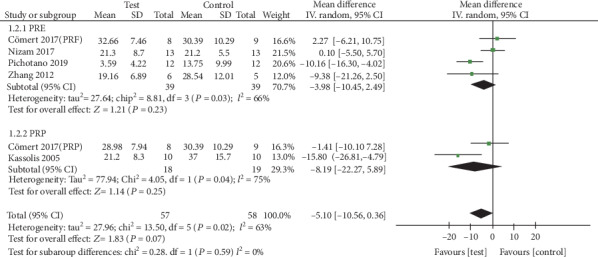
Percentage of residual bone substitute material (%).

*(2) Percentage of Residual Bone Substitute Material*. The data for residual bone substitute material were extracted from five studies [14, 16, 17, 20, 24]. The random effects model (RE) was employed (*I*^2^ = 63%). The results showed that the use of APCs determined no significant gain of residual bone substitute material when added to osteoconductive materials during the maxillary sinus augmentation (Figure 4), with an MD of -5.10% (95% CI, -10.56% to 0.36%; *p* = 0.07), and there were also no statistical improvement between the test and control in the subgroup of PRF and PRP, with an MD of -3.98% (95% CI, -10.56% to 0.36%; *p* = 0.23) and an MD of -8.19% (95% CI, -22.27% to 5.89%; *p* = 0.25).

**Figure 5:**
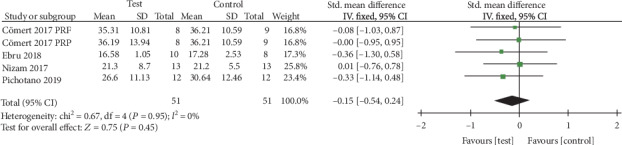
Percentage of soft tissue area.

*(3) Percentage of Soft Tissue Area*. The meta-analysis of soft tissue area was conducted among all the four studies [14–17]. The fixed effects model (FE) was applied (*I*^2^ = 0). The result of our meta-analysis showed no significant difference concerning the percentage of soft tissue area between the groups, with an SMD of 0.15% more soft tissue area observed in the APC group compared with the control group (95% CI, -0.54% to 0.24%; *p* = 0.45) (Figure 5).

#### 3.2.2. Implant Stability Quotient (ISQ) Values

**Figure 6:**
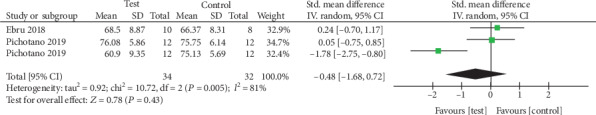
The implant stability quotient values (ISQ).

The implant stability quotient values (ISQ) were found in 2 articles [14, 15] with 3 periods in the two groups compared. And the random effects model (RE) was adopted owing to the high heterogeneity (Figure 6). The ISQ at implant loading (76.08 ± 5.86) was significantly higher than that after implant placement in the test group (60.9 ± 9.35); the results of the included studies showed that the use of PRF determines a greater ISQ than osteoconductive materials alone, but there was no statistically significant difference between the two groups (-0.48, 95% CI, -1.68 to 0.72; *p* = 0.43),

#### 3.2.3. Radiographic Evaluation

**Figure 7:**
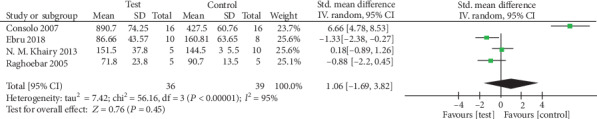
The bone density by radiological analysis.

Four studies [15, 19, 22, 23] reported the postoperative radiographic evaluation, and due to the high heterogeneity (*I*^2^ = 95%), a random effects model was selected. There was no significant difference concerning the bone density by radiological analysis between the groups, with an SMD of 1.06% less bone density observed in the APCs added to osteoconductive materials compared with the osteoconductive materials alone (1.06%, 95% CI, -1.69% to 3.82%; *p* = 0.45). The details of each study can be found in Figure 7.

### 3.3. Sensitivity Analysis

Sensitivity analyses were carried out by discarding one research every time to assess the impact of single research on the general outcomes. The overall stability of our results is shown in Table 3.

## 4. Discussion

In the past several decades, APCs have attracted the attention of scholars as a potential regenerative material in the treatment of tissue healing [25]. There were lots of researches on improving sinus augmentation with the use of regeneration materials [26–29], but there was a lack of meta-analysis on these clinical results. The present meta-analysis was aimed at evaluating the additional effect of APCs with osteoconductive materials on the sinus augmentation.

Generally, the histologic evaluation of sinus augmentation was mainly reflected in the percentage of new bone formation, percentage of residual bone substitute material, and percentage of soft tissue area. The results showed that the quantities of histologic evaluation were equivalent between the APC group and the non-APC group, and the result was similar to the findings of Liu's analysis [30], which indicated that there were also no statistical differences in new bone formation and newly formed bone substitute between the non-PRF and PRF groups. Besides, in the research of Pichotano et al. [14], the healing time between sinus augmentation and implant placement could be considerably reduced to 2-4 months by using PRF. The autogenous bone combined with PRP could also achieve the same favorable results based on the research report of Thor [21]. Recent studies [10] also showed similar results that adding PRF to the graft materials used for the maxillary sinus lifting had no beneficial effect on regeneration and new bone formation. These studies could not confirm the potential effect of PRF in promoting bone formation due to the long graft healing time [8, 26].

The radiological analysis of bone density was conducted on four studies, three of which were in PRP group [19, 22, 23] and only one in PRF group [15]. Olgun and Consolo reported the bone density of 86.66 ± 43.57 and 890.7 ± 74.25 at four months after grafting in APC group, respectively, compared to 160.81 ± 63.65 and 427.5 ± 60.76 at six months after grafting in the control group. Khairy showed the bone density was 151.5 ± 37.8 in sinus lifted by autogenous bone with PRP at 3 months and 144.5 ± 35.5 at 3 months after implantation in control group. However, there was no significant difference in sinus floor elevating between the APC group at about 3 months and the control group at about 6 months after grafting, indicating that APCs might influence the early bone healing even though they had no potential effect of accelerating bone formation in the long term. This result was confirmed by the animal studies of Miron and Gerard [31, 32]. There was no direct correlation between the application of APCs in sinus augmentation and the radiologic and histological variables, which might be due to the small size of RCTs.

In general, dental implant is placed in the maxillary sinus after 8 months of the floor augmentation healing, and the implant stability quotient (ISQ) was used to detect the contact of bone-to-implant that is important to the effect of sinus augmentation. Pichotano et al. [14] claimed that compared with the control group, the PRF plus DBBM group installed the implant 4 or 8 months after augmentation, and such a shorter healing time for implant played a vital role in increasing the implant stability. It was also found by Pichotano et al. that the sinus augmentation with DBBM plus PRF could shorten the time required for bone graft maturation and was conducive to the earlier implant placement. The study of Cömert Kılıç et al. [17] also proved that the addition of L-PRF improved implant stability and allowed for faster osseointegration, and ISQ values at loading demonstrated a significant increase in the test group compared to the initial value at implant placement (60.90 ± 9.35 and 76.08 ± 5.86). Through the histologic examination of the mini-implants retrieved, Aimetti et al. [33] found that at the abutment connection, a higher bone-to-implant contact rate was observed on the autogenous bone added with PRP than on the autogenous bone alone despite of similar clinical and radiographic healing patterns (46.75% ± 13.6% vs. 20.5% ± 5.57%, respectively). However, there was no statistically significant difference between the two groups in the result.

Sensitivity analyses were performed to detect the heterogeneity by the method of discarding research. The heterogeneity of histological assessment among these studies showed no significant difference except the percentage of soft tissue area (*I*^2^ = 0). Furthermore, APCs had no significant effect on the maxillary sinus elevation in the long term. The sensitivity analyses indicated the heterogeneity of the implant stability quotient values (*I*^2^ = 0%) existed between the studies due to the period of implant placement by discarding the study of Pichotano et al. [14] (implant placement 4 or 8 months after augmentation). The ISQ of the test and control groups who underwent implant placement 4 and 8 months after sinus augmentation, respectively, was measured. The results showed significantly higher ISQ values after implant placement in the control group (75.13 ± 5.69) than those in the test group (60.90 ± 9.35), but a significant increase in ISQ was observed in the test group at the time of implant loading (60.9 ± 9.35 to 76.08 ± 5.86, *p* = 0.0014). The above results might be attributed to sinus augmentation with PRF shortening the time required for implant healing, which agree with Canellas [34]. In their systematic review, they showed PRF increased implant stability 1 week and 1 month after implant surgery and no difference between the use of PRF mixture with bone substitute or bone substitute alone in sinus lift procedure.

Concentrated growth factors (CGFs) are the PRF derivatives developed by Rodella et al. in 2006, and there is a much larger, denser, and richer growth factor fibrin matrix in CGFs than PRF [11]. In our analysis, CGFs are not present in subgroups for lack of randomized controlled studies, but some other studies indicated its importance in sinus floor augmentation. A study on canine model aiming at assessing the effect of Bio-Oss plus concentrated growth factors (CGFs) on bone regeneration for maxillary sinus floor augmentation showed that grafting with Bio-Oss in combination with CGFs could increase new bone formation more efficiently than using Bio-Oss alone [35]. Autologous fibrin-rich blocks with concentrated growth factors (CGFs) were found in a retrospective study to be the predictability of new bone formation in both the maxillary sinus augmentation and implant placement. Anitua et al. reported that autologous platelet aggregates [36], such as platelet-rich plasma and platelet-rich fibrin gel in CGFs, have been used to accelerate new bone formation associated with guided bone regeneration and sinus grafting for many years.

At present, there are several meta-analyses on APCs for alveolar ridge preservation, gingival recession, and tooth extraction. However, there is less analysis on APCs for sinus augmentation and just few systematic reviews about PRF or PRP on maxillary sinus augmentation [37–39] and one meta-analysis [30] on PRF. In their reviews, PRF or PRP had effect on soft tissue healing and postoperative symptomatology, but it was conflicting on new bone formation during maxillary sinus augmentation when combined with other biomaterials. Similar to the results of this study, the percentages of residual bone substitute material and new bone formation were no significant difference between APC group and non-APC group in our results. There are more effective analyses to the subgroup of APC group and less postoperative symptomatology analysis in our research, compared with Liu's analysis and other reviewers.

However, there were some limitations to this meta-analysis. Firstly, the inherent heterogeneity could not be avoided between the included articles, 7 articles were split-mouth design and 4 articles were parallel design, which affect the methodological analysis. Secondly, only PRF and PRP subgroup analysis and lacking RCTs of CGFs were enrolled in this analysis, which may have reduced the power in detecting significant differences. Lastly, limitation of this paper was the high risk of bias in the selected studies. Because of the methodological shortcomings for the production processes of APCs, it was impossible to conduct the allocation concealment strictly or to be blind to the personnel in the surgery. Our risk of bias assessment results showed that among 11 included studies, 5 studies were classified as moderate risk and 6 as high risk. Taken together, the conclusions of our analysis are limited, and more studies with low risk of bias in this field are needed in the future.

## 5. Conclusion

According to the system review and meta-analysis, we can initially get the following conclusions:

1. Based on the limited studies, it seemed that PRF or PRP failed to show additional effect on new bone formation and implant stability when combined with osteoconductive materials. It is not currently recommended for routine use PRF or PRP as an osteoinductive material to bone grafting in sinus augmentation
2. The addition of APCs to osteoconductive materials in sinus augmentation may help to reduce the healing time and postoperative symptomatology and conducive to shortening the time required for bone graft maturation and allows earlier implant placement, but it need more RCTs to confirm
3. The limited RCTs for CGFs and high risk of bias of studies and more low-risk correlational studies were needed

## Figures and Tables

**Table:** (a) Characteristics of the included studies

References (year)	Study design (duration)	Country	No. of patients (sinus)	Intervention (no. of patients)	Sex (no. of patients)	Age (years)
Nizam et al. (2018) [16]	RCT/a split-mouth (12 months)	Turkey	13 (26)	TG: 13/CG: 13	4 F/9 M	49.92 ± 10.37
Olgun et al. (2018) [15]	RCT/parallel (6 months)	Turkey	18 (18)	TG: 10/CG: 8	9 F/9 M	42-69
Cömert Kılıç et al. (2017) [17]	RCT/parallel (6 months)	Turkey	26 (26)	TG: 17/CG: 9	9 F/17 M	22-51
Taschieri et al. (2016) [18]	RCT/a split-mouth design (6 months)	Italy	5 (10)	TG: 5/CG: 5	3 F/2 M	48-71
Zhang et al. (2012) [20]	RCT/parallel (6 months)	China	10 (11)	TG: 6/CG: 5	2 F/8 M	30-53
Kassolis and Reynolds (2005) [24]	RCT/a split mouth (6 months)	U.S	10 (20)	TG: 10/CG: 10	NR	22-58
Khairy et al. (2013) [19]	RCT/parallel (6 months)	Egypt	15 (15)	TG: 10/CG: 5	NR	22-54
Pichotano et al. (2019) [14]	RCT/a split-mouth (8 months)	Brazil	12 (24)	TG: 12/CG: 12	6 F/6 M	43-63
Thor et al. (2007) [21]	RCT/a split-mouth (6 months)	Sweden	11 (22)	TG: 11/CG: 11	10 F/1 M	36-72
Raghoebar et al. (2005) [23]	RCT/a split-mouth (20.2 ± 4.3 months)	The Netherlands	5 (10)	TG: 5/CG: 5	3 F/2 M	57-62
Consolo et al. (2007) [22]	RCT/a split-mouth (4-7 months)	Italy	16 (32)	TG: 16/CG: 16	11 F/5 M	37-57

**Table:** (b) Characteristics of the included studies

References (year)	APC preparation, the kind and use of the APCs	Surgical procedure	Complications and prognosis
Nizam et al. (2018) [16]	Peripheral blood, 400×g/12 min, L-PRF membrane cut into fragments to mix with DBBM, L-PRF, and membrane	TG: the maxillary sinus augmentation procedure was randomly performed using DBBM +L-PRF mixture. CG: the maxillary sinus augmentation procedure was randomly performed using DBBM alone	A significant bleeding with control

Olgun et al. (2018) [15]	T-PRF: the blood (20 ml) is centrifuged in titanium tubes (878 g, 12 minutes), PRF membrane	TG: to augment the sinus floor by T-PRF alone and implant after 4 months CG: to augment the sinus floor by allografts alone and implant after 6 months	No

Cömert Kılıç et al. (2017) [17]	PRP: 10 ml blood with anticoagulant, 3000 rpm/10 min. classification of Donhan Ehrenfest PRF: 10 ml blood, 3000 rpm/10 min, PRF membrane	TG: the maxillary sinus-floor elevation with PRF+ *β*-TCP mixture grafts or the maxillary sinus-floor elevation with P-PRP+ *β*-TCP mixture grafts. CG: the maxillary sinus-floor elevation with *β*-TCP alone.	5 sinus perforations with collagen membrane covered

Taschieri et al. (2016) [18]	580g/8 min, a P-PRP gel	TG: The maxillary sinus floor augmentation surgery with lateral window approach with DBBM and PRP CG: The maxillary sinus floor augmentation surgery with lateral window approach with DBBM alone	No

Zhang et al. (2012) [20]	300 g for 10 min, PRF membrane and fragment	TG: The maxillary sinus floor augmentation surgery by the lateral wall with Bio-Oss and PRF CG: The maxillary sinus floor augmentation surgery by the lateral wall with Bio-Oss alone	No

Kassolis and Reynolds (2005) [24]	80 ml blood, centrifuged for approximately 1 minute and separating the red cell component at the bottom result in PRP	TG: maxillary sinus augmentation with FDBA+PRF CG: maxillary sinus augmentation with FDBA	No

Khairy et al. (2013) [19]	20 ml blood for PRP, 5600 rpm/15 min and 2400 rpm/10 min. combined with the corticocancellous particulate into the elevated sinus.	TG: maxillary sinus augmentation with autogenous bone+PRF and implant insertion at4 or 6 months CG: maxillary sinus augmentation with autogenous bone and implant insertion at 6 months	5 patients of sinus perforation with control

Pichotano et al. (2019) [14]	20 ml blood, 300 g/10 min, L-PRF membrane	TG: the bilateral maxillary sinus augmentation with DBBM +PRF and implant insertion at 4 months CG: the bilateral maxillary sinus augmentation with DBBM and implant insertion at 6 months	No

Thor et al. (2007) [21]	450 ml blood of whole blood from a peripheral vein of the arm or foot, 5600 rpm and 2400 rpm for PRP	TG: the left side of maxillary sinus augmentation with particulated autogenous bone + PRP and implant insertion at 3 months CG: he left side of maxillary sinus augmentation with particulated autogenous bone alone and implant insertion at 3 months	No

Raghoebar et al. (2005) [23]	60 ml blood of whole blood with the TGF-*β* concentration according to the method described by Waarde for PRP	TG: the one side of maxillary sinus augmentation with autogenous bone +PRP and implant insertion at 3 months CG: the left side of maxillary sinus augmentation with autogenous bone alone and implant insertion at 3 months	One implant with removed and one sinus perforation with healing

Consolo et al. (2007) [22]	450 ml blood of whole blood with container containing an anticoagulant for PRP, 1200 g/6 min at 20°C and 4400 g/6 min at 14°C.	TG: the one side of maxillary sinus augmentation with autogenous bone +PRP and implant insertion at 4-7 months CG: the other side of maxillary sinus augmentation with autogenous bone alone and implant insertion at 4-7 months	No

CG: control group; TG: test group; L-PRF: leukocyte-and platelet-rich fibrin (L-PRF); DBBM: deproteinized bovine bone mineral; NR: not report.

**Table 2:** Outcome date for the included studies.

References (year)	Outcome	
	Significant:	Nonsignificant:
Nizam et al. (2018) [16]	NR	(1). Augmented (C: 2.53 ± 0.61, T: 2.45 ± 0.79) and residual bone height (C: 13.53 ± 1.20, T: 13.60 ± 1.09) (2). The percentage of newly formed bone (C: 21.2 ± 5.5, T: 21.3 ± 8.7) (3). The percentage of soft tissue component (C: 45.9 ± 8.3%, T: 52.6 ± 12.5%)

Olgun et al. (2018) [15]	Bone volume, density, and height values were significantly higher in the allografts alone group than T-PRF alone group	(1). The ISQ in T-PRF group (68.50 ± 8.87) at 4 months and control group (66.37 ± 8.31) at 6 months. (2). The rate of newly formed bone in T-PRF group (16.58 ± 1.05) at 4 months and control group (17.28 ± 2.53) at 6 months. (3). The cancellous bone ratio in T-PRF group (24.00 ± 1.50) at 4 months and control group (22.69 ± 2.63) at 6 months.

Cömert Kılıç et al. (2017) [17]	Osteoprogenitor cells (0.042 ± 0.01/1000 *μ*m^2^) were lower and inflammatory cells (0.043 ± 0.01/1000 *μ*m^2^) were higher in the PRF group	The new bone formation; mean percentages of residual graft; the mean percentages of soft tissue; mean densities of osteoblasts, osteoclasts, osteocytes, and capillary vessels; and the composition and distribution of histologic structures

Taschieri et al. (2016) [18]	NR	(1). The mean percentage of vital bone (%) at 6th month. DBBM+P-PRP: 30.7 ± 7.89, *β*-TCP: 22.72 ± 9.21 (2).The mean residual bone height (mm) at 6th month DBBM+P-PRP: 2.80 ± 1.04, *β*-TCP: 2.40 ± 1.08

Zhang et al. (2012) [20]	There were no obvious signs of resorption by the postoperative radiographic evaluation in both groups	(1). The percentage of newly formed bone (%) at 6th month. Bio-Oss + PRF group: 18.35 ± 5.62, Bio-Oss group: 12.95 ± 5.33 (2). The percentage of residual bone substitute (Bio-Oss) (%) at 6th month, Bio-Oss + PRF group: 19.16 ± 6.89, Bio-Oss group: 28.54 ± 12.01 (3). Contact between newly formed bone and bone substitute (%). Bio-Oss + PRF group: 21.45 ± 14.57, Bio-Oss group: 18.57 ± 5.39

Kassolis and Reynolds (2005) [24]	(1). A significantly greater percentage of vital tissue (bone and connective tissue) in TG (2). Higher proportion of the regenerate after grafting with FDBA and PRP (3). The ratio of vital bone to residual graft particles in TG was higher than CG	The vertical dimension

Khairy et al. (2013) [19]	(1). A significant increase in mean bone density for TG immediately, at 3 months and 6months (2). Significant highest mean bone density for TG at 6 months postimplantation	The mean bone density of grafting in CG at 3 months The difference of mean bone density for grafting at 3 months between both groups

Pichotano et al. (2019) [14]	(1). Histological evaluation demonstrated increased percentage of newly formed bone (%) for the TG (44.58 ± 13.9) compared to the CG (30.02 ± 8.42). (2). Residual graft (%) was significantly higher in the CG (13.75 ± 9.99) than in TG (3.59 ± 4.22) (3). The ISQ immediately after implant placement was significantly higher in the control group (75.13 ± 5.69) compared to the test group (60.9 ± 9.35).	(1). There was not differences in graft volume between test and control group by cone-beam computed tomography analysis (2). The ISQ values at loading did not differ between the groups
Thor et al. (2007) [21]	Histological evaluation with 7 patients demonstrated increased percentage of newly formed bone (%) for the TG (22 ± 9) compared to the CG (11 ± 3) at 3 months	There were no differences in percentage of newly formed bone with 9 patients between test and control group by histological evaluation of biopsies with microimplants retrieved 6 months after bone grafting (TG vs. CG: 14% ± 7% vs. 13% ± 6%)

Raghoebar et al. (2005) [23]	NR	(1).The average density on the microradiographs at the first molar region was at the PRP side 71.8 ± 23.8, and at the non-PRP side 90.7 ± 13.5 (2). The newly bone formation by the histomorphometric analysis revealed no differences between both groups (TG vs. CG: 38.4% ± 11.3% vs. 41.1% ± 8.3%)

Consolo et al. (2007) [22]	(1). Both of groups showed an almost uniform radiographic aspect (2). Densitometric values were higher at PRP group (at 4 months TG vs. CG: 890.7HU ± 74.25HU vs. 522.9 HU ± 65.73HU; at 5 months TG vs. CG: 820HU ± 67.82HU vs. 462.6 HU ± 62.88HU; at 6 months TG vs. CG: 626.2HU ± 97.07HU vs. 427.5 HU ± 60.76HU; at 7 months TG vs. CG: 500HU ± 40.82HU vs. 392.5 HU ± 61.85HU) (3). Histology documents enhanced bone activities by trabecular bone volume (TBV) evaluations in sites treated with PRP group at 4 and 5 months (at 4 months TG vs. CG: 43.3 ± 9.1 vs. 26 ± 5.2; at 5 months TG vs. CG: 39.3 ± 5.7 vs. 29.2 ± 4)	(1). Clinical performance across both groups showed no statistical significance (2). The TVB values of PRP group and autologous bone alone group at 6 and 7 months

CG: control group; TG: test group; L-PRF: leukocyte-and platelet-rich fibrin (L-PRF); DBBM: deproteinized bovine bone mineral; NR: not report; T-PRF: titanium-prepared platelet rich fibrin; *β*-TCP: beta-tricalcium phosphate; P-PRP: pure platelet-rich plasma; ISQ: implant stability quotient; TBV: trabecular bone volume.

**Table:** (a) Percentage of new bone formation

The discarding study	WMD (95% CI)	Heterogeneity (*I*^2^, %)
Thor et al. 2007 [21]	1.87 (-2.14, 5.89)	71
Cömert Kılıç et al. 2017 (PRF) [17]	3.44 (-1.09, 7.96)	79
Cömert 2017 (PRP) [17]	3.12 (-1.34, 7.58)	79
Ebru 2018	3.60 (-1.54, 8.74)	75
Kassolis and Reynolds 2005 [24]	2.57 (-1.84,6.99)	78
Khairyet al. 2013 [19]	4.48 (-1.84,8.35)	71
Nizam 2017	3.38 (-1.38,8.14)	79
Pichotano et al. 2019 [14]	1.85 (-2.13,5.83)	73
Taschieri et al. 2016 [18]	2.56 (-1.78,6.89)	78
Zhang et al. 2012 [20]	2.69 (-1.85,7.23)	78

**Table:** (b) Percentage of residual bone substitute material

The discarding study	WMD (95% CI)	Heterogeneity (*I*^2^, %)
Cömert Kılıç et al. (PRF) [17]	-6.56 (-12.49, -0.63)	63
Cömert Kılıç et al. (PRP) [17]	-5.93 (-12.42, 0.57)	69
Kassolis and Reynolds 2005 [24]	-3.48 (-8.68, 1.72)	56
Nizam 2017	-6.53 (-12.78, -0.31)	59
Pichotano et al. 2019 [14]	-3.71 (-9.57, 2.15)	56
Zhang et al. 2012 [20]	-4.55 (-10.66,1.55)	69

**Table:** (c) The Implant stability quotient values

The discarding study	SMD (95% CI)	Heterogeneity (*I*^2^, %)
Ebru 2018	-0.84 (-2.63, 0.95)	88
Pichotano et al. 2019 [14] (implant loading)	-0.77 (-2.74, 1.20)	88
Pichotano et al. 2019 [14] (implant immediately)	0.13 (-0.48, 0.74)	0

**Table:** (d) The bone density by radiological analysis

The discarding study	SMD (95% CI)	Heterogeneity (*I*^2^, %)
Consolo et al. 2007 [22]	-0.67 (-1.60, 0.27)	50
Ebru 2018	1.92 (-1.88, 5.71)	96
Khairy et al. 2013 [19]	1.42 (-2.80, 5.63)	96
Raghoebar et al. 2005 [23]	1.75 (-2.03, 5.53)	96
